# Supplementary material for: Apical Sperm Hook Morphology Is Linked to Sperm Swimming Performance and Sperm Aggregation in Peromyscus Mice
Source: Cells. 2021 Sep 1;10(9):2279. doi: 10.3390/cells10092279 (PMC8471468; doi:10.3390/cells10092279)
Supplement: Supplementary file 1 [file cells-10-02279-s001.zip › cells-1269214-supplementary.pdf]

Apical sperm hook morphology is linked to sperm swimming performance and sperm aggregation in *Peromyscus* mice

Kristin A. Hook, Lauren M. Wilke and Heidi S. Fisher

Supplementary information

Table S1. Mean (± SE) measurements for linear and relative sperm hook morphology traits within six closely related focal species of *Peromyscus* mice

***Peromyscus* Species**	**Sperm Trait**					
	**Linear Measures**			**Relative Measures**		
	Hook Length (μm)	Hook Width (μm)	Hook Area (μm^2)	Relative Hook Length	Relative Hook Width	Relative Hook Area
P. californicus	4.22 ± 0.05 (3.8%)	0.61 ± 0.01 (3.9%)	1.46 ± 0.02 (6.2%)	0.90	0.22	0.12
P. eremicus	4.78 ± 0.04 (3.9%)	0.71 ± 0.01 (4.1%)	1.88 ± 0.03 (6.3%)	0.97	0.22	0.13
P. polionotus	4.23 ± 0.03 (4.3%)	0.65 ± 0.01 (4.3%)	1.53 ± 0.02 (6.7%)	0.93	0.21	0.12
P. maniculatus	4.20 ± 0.04 (4.5%)	0.67 ± 0.01 (4.8%)	1.43 ± 0.02 (7.3%)	0.89	0.21	0.11
P. leucopus	4.07 ± 0.04 (4.6%)	0.63 ± 0.01 (5.8%)	1.39 ± 0.02 (7.9%)	0.89	0.23	0.12
P. gossypinus	4.40 ± 0.04 (6.0%)	0.64 ± 0.01 (6.5%)	1.42 ± 0.03 (8.7%)	0.94	0.23	0.12

Relative hook measures are based on mean head length, head width, and head area, respectively. Coefficients of variation for each linear trait are indicated within parentheses.
